# A Manual of Diseases of the Nervous System

**Published:** 1892-09

**Authors:** 


A Manual of Diseases of the Nervous System. By W. R. Gowers, M. D., F. R C. P., F. R. S., Consulting Physician to the University College Hospital; Physician to the National Hospital for the Paralyzed and Epileptic. Second edition. Revised and enlarged. Volume I. Diseases of the Nerves and Spinal Cord, with 180 illustrations, including 370 figures. Pp. 616. Cloth. Philadelphia: P. Blakiston, Son & Co., 1012 Walnut street. 1892.

The excellence of the first edition of Gowers's work won for it at once a high place as an authority. So careful has the author been in explanation and elaboration of his work, that he easily takes rank as one of the clearest writers it has ever been our pleasure to read.

The second edition has been thoroughly revised, and new chapters added. The work is fully up to date, and we can best express our opinion of it by saying that there is no better work on nervous diseases, and very few so good and complete. The publishers have done their work in the very best style. The type, paper, and illustrations, all are excellent. J. W. P.

